# Mining and state-space modeling and verification of sub-networks from large-scale biomolecular networks

**DOI:** 10.1186/1471-2105-8-324

**Published:** 2007-08-31

**Authors:** Xiaohua Hu, Fang-Xiang Wu

**Affiliations:** 1 College of Information Science & Technology, Drexel University, Philadelphia, PA 19104, USA; 2 Department of Mechanical Engineering, University of Saskatchewan, Saskatoon, SK, S7N 5A9, Canada; 3 Division of Biomedical Engineering, University of Saskatchewan, Saskatoon, SK, S7N 5A9, Canada

## Abstract

**Background:**

Biomolecular networks dynamically respond to stimuli and implement cellular function. Understanding these dynamic changes is the key challenge for cell biologists. As biomolecular networks grow in size and complexity, the model of a biomolecular network must become more rigorous to keep track of all the components and their interactions. In general this presents the need for computer simulation to manipulate and understand the biomolecular network model.

**Results:**

In this paper, we present a novel method to model the regulatory system which executes a cellular function and can be represented as a biomolecular network. Our method consists of two steps. First, a novel scale-free network clustering approach is applied to the large-scale biomolecular network to obtain various sub-networks. Second, a state-space model is generated for the sub-networks and simulated to predict their behavior in the cellular context. The modeling results represent *hypotheses *that are tested against high-throughput data sets (microarrays and/or genetic screens) for both the natural system and perturbations. Notably, the dynamic modeling component of this method depends on the automated network structure generation of the first component and the sub-network clustering, which are both essential to make the solution tractable.

**Conclusion:**

Experimental results on time series gene expression data for the human cell cycle indicate our approach is promising for sub-network mining and simulation from large-scale biomolecular network.

## Background

We are in the era of holistic biology. Massive amounts of biological data await interpretation. This calls for formal modeling and computational methods. In this paper, we present a method to model the regulatory system which executes a cellular function and can be represented as a *biomolecular network*. Understanding the biomolecular network implementing some cellular function goes beyond the old dogma of "one gene: one function": only through comprehensive system understanding can we predict the impact of genetic variation in the population, design effective disease therapeutics, and evaluate the potential side-effects of therapies. As biomolecular networks grow in size and complexity, the model of a biomolecular network must become more rigorous to keep track of all the components and their interactions. In general this presents the need for computer simulation to manipulate and understand the biomolecular network model. However, a major challenge of modeling the dynamics of a biomolecular network is that conventional methods based on physical and chemical principles (such as systems of differential equations) require data that are difficult to accurately and consistently measure using either conventional or high-throughput technologies, which characteristically yield noisy, semi-quantitative, and often relative data.

In this paper, we present a hybrid approach that combines data mining and state-space modeling to build and analyze the biomolecular network of a cellular process. Our method consists of two steps. First, a novel scale-free network clustering approach is applied to the large-scale biomolecular network to obtain various sub-networks. Second, a state-space model is generated for the sub-networks and simulated to predict their behavior in the cellular context. It integrates the process of obtaining network structure directly with state-space dynamic simulation robust to qualitative (molecular biology) and noisy quantitative (biochemical) data to iteratively test and refine hypothetical biomolecular networks. In the following, we review some related work in community structure analysis, and biomolecular networking modeling.

### Community structure analysis

The study of community structure in a network is closely related to the graph partitioning in graph theory and computer science. It has also closely ties with the hierarchical clustering in sociology [1]. Recent years have witnessed an intensive activity in this field, partly due to the dramatic increase in the scale of networks being studied. Because communities are believed to play a central role in the functional properties of complex networks [1], the ability to detect communities in networks could have practical applications. Studying the community structure of biological networks is of particular interest and challenging, given the high data volume and the complex nature of interactions. In the context of biological networks, communities might represent structural or functional groupings. They can be synonymous with molecular modules, biochemical pathways, gene clusters, or protein complexes. Being able to identify the community structure in a biological network may help us to understand better the structure and dynamics of biological systems. Hashimoto and colleagues [2] have developed an approach to growing genetic regulatory networks from seed genes. Their work is based on probabilistic Boolean networks and sub-networks are constructed in the context of a directed graph using both the coefficient of determination and the Boolean function influence among genes. The similar approach is also taken by Flake and colleagues [3] to find highly topically related communities in the Web based on the self-organization of the network structure and on a maximum flow method. Related works also include those that predict co-complex proteins. Jansen and colleagues [4] use a procedure integrating different data sources to predict the membership of protein complexes for individual genes based on two assumptions: first, the function of any protein complex depends on the functions of its subunits; and second, all subunits of a protein complex share certain common properties. Bader and Hogue [5] report a molecular complex detection (MCODE) clustering algorithm to identify molecular complexes in a large protein interaction network. MCODE is based on local network density – a modified measure of the clustering coefficient. Bu and colleagues [6] use a spectral analysis method to identify the topological structures such as quasi-cliques and quasi-bipartites in a protein-protein interaction network. These topological structures are found to be biologically relevant functional groups. In our previous work, we developed a spectral-based clustering method using local density and vertex neighborhood to analyze the chromatin network [7,8]. Two recent works along this line of research are based on the concept of network modularity introduced by Hartwell and colleagues [9]. The works by both Spirin and Mirny [10] and Rives and Galitski [11] use computational analyses to cluster the yeast PPI network and discover that molecular modules are densely connected with each other but sparsely connected with the rest of the network.

### Biomolecular networking modeling

A variety of approaches to state models have been implemented for gene and protein networks, including among others, hidden Markov models [12,13], Bayesian networks [14-16], linear networks [17-19], finite state [20], and probabilistic Boolean networks [21,22]. These and other methods are based on either treating biological variables at the crudest resolution (on or off in Boolean networks, a few more levels possible for finite state models but with rapidly growing complexity) or as absolute physical quantities. Boolean networks [23] are computationally simple and do not depend on precise experimental data, and thus they are potentially suitable for handling both the complexity of biological networks and qualitative text-based data. However, Boolean models have been proven to lack the resolution needed to accurately model biomolecular interactions [24]. In contrast, various differential equation-based models [17,18,25] are computationally expensive and sensitive to imprecisely measured parameters (and virtually useless given purely qualitative data, i.e. from text-mining). Fuzzy logic [26] provides a mathematical framework that is compatible with poorly quantitative yet qualitatively significant data, but it tends to generate so many rules used to describe biological systems [27].

## Results

**Figure 1:**
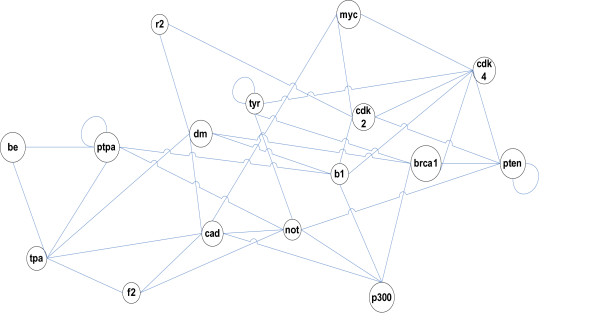
A sub-network of human genes.

To evaluate the accuracy and feasibility of state-space biomolecular network modeling, we considered the gene network corresponding to a sub-network found using *SNBuilder *proposed in this paper. The sub-network as shown in Figure 1 involves human genes related to p53, apoptosis, DNA damage response, and cell cycle. Edges in Figure 1 are taken to represent potential connections between genes, defining the structure of the gene network. Table 1 shows the genes that encode the proteins in Figure 1. This results in some differences in terminology; for example, EP300 encodes the protein p300. Also, where aliases for gene names exist, the more common usage is given in Table 1.

**Table 1:** Algorithm: SNBuilder(G, s, f, d)

**Gene name**	DMTF	BRCA1	HIFX	HE	PPP2R4	MYC	NR4A2	F2
**Protein Name**	dm	brca1	h1	he	ptpa	myc	not	F2
**Gene name**	PTEN	RRM2	PLAT	TYR	CAD	CDK2	CDK4	EP300
**Protein Name**	pt	R2	tpa	tyr	cad	cdk2	Cdk4	P300

In this paper, we employ the human cell cycle gene expression data [28] to construct the state-space model of the sub-network shown in Figure 1. There are independent data in [28] for five methods of cell cycle synchronization, and out of which two datasets "Thy-Thy3" and "Thy-Noc" are complete for the genes in the sub-network we studied. These two datasets are swapped and used as the training dataset and the testing dataset in the two experiments.

As the human cell cycle gene expression data are very noisy, some data preprocessing techniques are applied to the log-ratio gene expression data. Firstly a filter is applied to gene expression profiles one by one. At a given time point, the new expression value is the average of three raw values at the immediately previous, current and immediatly behind points. As the mean values and magnitudes for genes and microarrays mainly reflect the experimental procedure [29], then the expression profile of each gene is normalized to have the mean of zero and the standard deviation of one, and then for the expression values on each microarray as so to have the median of zero and the standard deviation of one. Such normalizations also make the PPCA simple [30].

### Experiment 1

**Figure 2:**
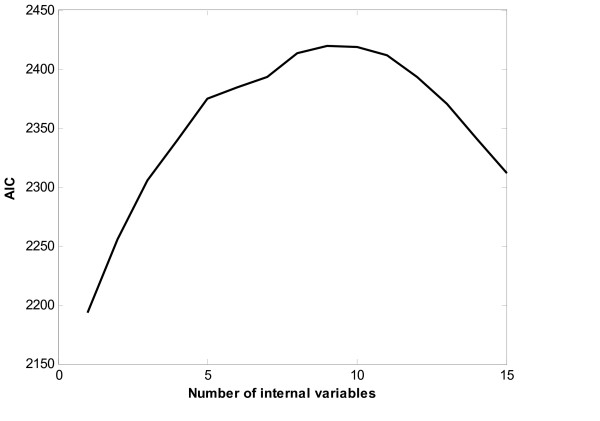
Plots of AIC with respect to the number of internal variables in the Thy-Thy3.

This experiment treats the Thy-Thy3 as the training dataset and the Thy-Noc as the testing dataset. Figure 2 depicts the profile of AIC with respect to the number of internal variables in the Thy-Thy3 dataset. Using the method presented in the section Method, the number of internal variables is determined to be nine. The transformation matrix ***C ***is calculated by equation (5), and further the expression profiles of the internal state variables are calculated and collected in matrix ***Z ***by formula (8). Control matrix **B **is determined such that it maximizes the ratio of the squared sum of the elements of *CB *corresponding to non-zeros in S to that of the elements of CB corresponding to zeros in S. As the human cell cycle gene expression data are collected at the equally spaced time points. The least square method for the linear regression problem is applied to determine the elements of matrix ***A ***in model (1).

To inspect stability, robustness, and periodicity of the inferred gene networks, the eigenvalues of the state transition matrices ***A ***are calculated. The eigenvalues of matrix follow as: -0.0715, 0.2479, 0.9018, 0.6749 ± 0.3959i, 0.8125 ± 0.2924i, 1.0396 ± 0.1536i. All eigenvalues except for the last pair of matrix ***A ***lie inside the unit circle in the complex plane, and the last pair is very closed to the boundary of the unit circle. This means that the inferred network is almost stable and robust. Furthermore, the dominant eigenvalues of the inferred network are pairs of conjugate complex number: 1.0396 ± 0.1536i. Accordingly, this implies that the network behaves periodically [31]. This result is because the networks are inferred from cell-cycle regulated gene expression data.

**Figure 3:**
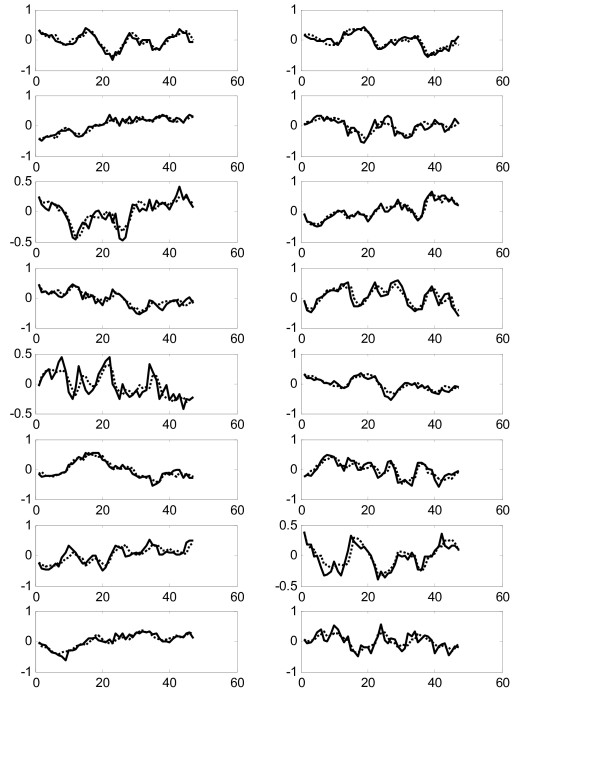
Comparison of experimental (solid lines) and predicted (dotted lines) gene expression profiles in the training data Thy-Thy3 in Experiment 1.

**Figure 4:**
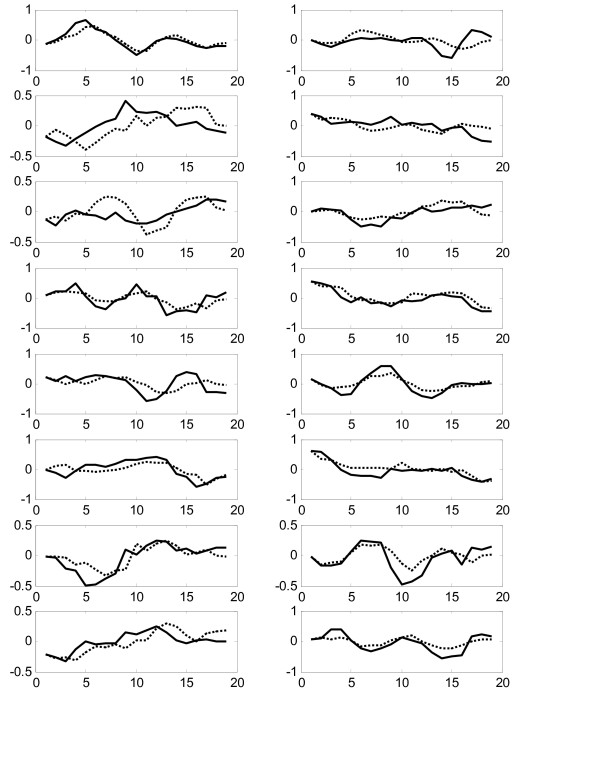
Comparison of experimental (solid lines) and predicted (dotted lines) gene expression profiles in the testing data Thy-Noc in Experiment 1.

Figure 3 shows comparison of all 16 experimental gene expression profiles and the predicted profiles from the constructed model on the training dataset "Thy-Thy3". The prediction error is calculated by equation (12) as 0.2525 and the prediction correlation is calculated as 0.8633, which is very good in agreement. We also use the constructed model to predict the expression profiles from the testing dataset "Thy-Noc". Figure 4 shows comparison of experimental and predicted expression profiles of all genes. The prediction correlation between the experiment and the predicted profiles for all genes in the testing dataset is calculated as 0.6623, which is less than that for the training dataset, but is still good as it is greater than 0.5.

### Experiment 2

**Figure 5:**
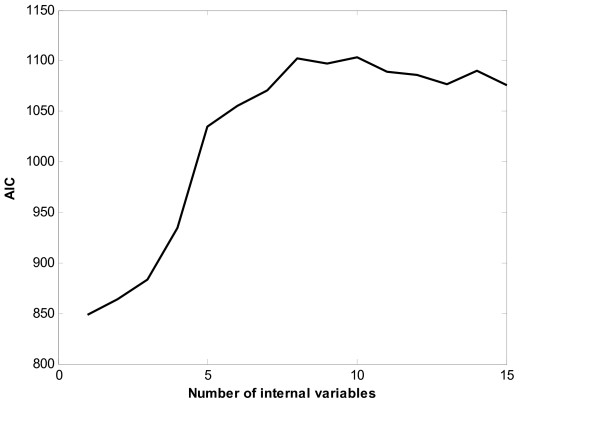
Plots of AIC with respect to the number of internal variables in the Thy-Noc.

Alternatively, this experiment treats the Thy-Noc as the training dataset and the Thy-Thy3 as the testing dataset. Figure 5 depicts the profile of AIC with respect to the number of internal variables in the Thy-NOC dataset, which indicate that the number of internal variables is eight. Using the proposed method in the section Method, matrices ***A***, ***B***, and ***C ***in model (1) are determined. Similar as in Experiment 1, the inferred network is almost stable and robust, and behaves periodically [31].

**Figure 6:**
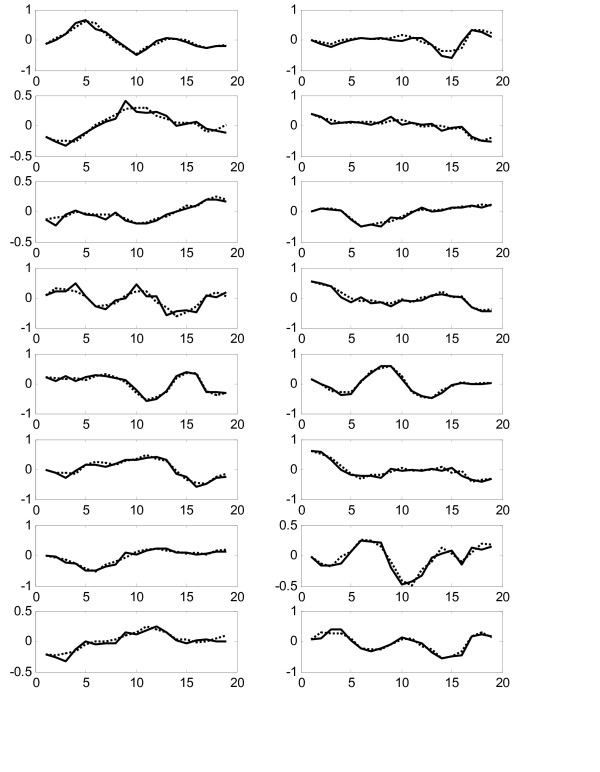
Comparison of experimental (solid lines) and predicted (dotted lines) gene expression profiles for the training data Thy-Noc in Experiment 2 (ER = 0.1095, RR = 0.9455).

**Figure 7:**
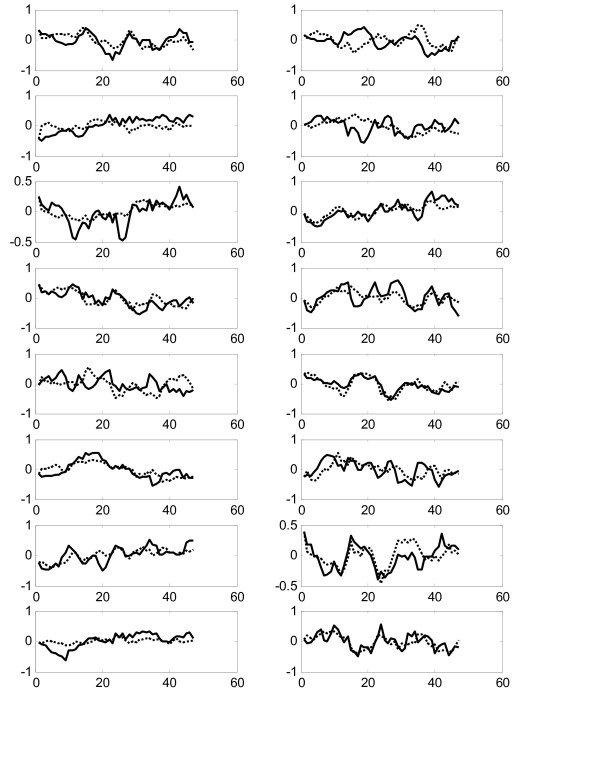
Comparison of experimental (solid lines) and predicted (dotted lines) gene expression profiles for the testing data Thy-Thy3 in Experiment 2 (ER = 0.8891, RR = 0.5159).

Figure 6 shows comparison of all 16 experimental gene expression profiles and the predicted profiles from the constructed model on the training dataset "Thy-NOC". The predicted error is calculated by equation (12) as 0.1095 and the average correlation is calculated as 0.9455, which is pretty good in agreement. We also use the constructed model to predict the expression profiles from the testing dataset "Thy-Thy3". Figure 7 shows comparison of experimental and predicted expression profiles of all genes. The prediction correlation between the experiment and the predicted profiles for all genes in the testing dataset is calculated as 0.5159, which is also good as it is greater than 0.5.

## Discussion and conclusion

In this paper, we present a new method for adaptive modeling of biomolecular networks. The biomolecular network model of cell function comprises gene and protein expression, interaction, and regulation. The method iteratively mines and organizes quantitative and qualitative data to generate scalable hypothetical biomolecular network structures. The dynamics of these computational hypotheses are tested and refined through cycles of simulation of state-space model and laboratory experiments. We use state-space modeling methods previously developed for gene networks as a robust and general representation for heterogeneous quantitative, qualitative, and linguistic biomolecular data. While in the example here only microarray data are presented, the state-space framework of representing biomolecular expression states can be simply extended to protein and metabolite levels. This is a key point, because gene networks are an abstraction representing only one aspect of biomolecular networks, and they must be integrated with protein-protein interaction networks, and metabolite profiling to develop a comprehensive portrait of cellular function.

We present in this paper an efficient approach to growing a community from a given seed protein. It uses topological property of community structure of a network and takes advantage of local optimization in searching for the community comprising of the seed protein. Due to the complexity and modularity of biological networks, it is more desirable and computationally feasible to model and simulate a network of smaller size. Our approach builds a community of manageable size and scales well to large networks. Its usefulness is demonstrated by the experimental results that all the four communities identified reveal strong structural and functional relationships among member proteins. It provides a fast and accurate way to find a community comprising a protein or proteins with known functions or of interest. For those community members that are not known to be part of a protein complex or a functional category, their relationship to other community members may deserve further investigation which in turn may provide new insights.

Although we do not explicitly use our approach to the prediction of co-complexed proteins, the results of comparing with the PNR method developed by Asthana and colleagues [32] have shown that the communities identified by our approach do include the top ranked candidates of co-complexed proteins. Our approach does not consider the quality of data in our downloaded data set. By using the strong sense definition of community [33], we could to some degree reduce the noises. However, to improve the quality of an identified community, we have to take into account the quality of data and that is another part of our future work. One possible way is to use the probabilities assigned to individual protein pairs as used by Jasen et al [4], Radicchi et al [33], and Bader et al [5,34].

In general, the state-space modeling method allows for inconsistencies and potentially noisy data to be identified and used to generate alternative computational hypotheses for biomolecular networks. The method is tractable and scalable because novel clustering methods are applied to adaptively extract biologically significant sub-networks for simulation and hypothesis testing. State space simulation of hypothetical biomolecular network models is compared with experimental data to select and refine plausible hypotheses. We combine the simulation result with the computationally derived meta-model to identify key genes whose perturbation would generate the data set that could most optimally differentiate between the alternative biomolecular network hypotheses. Consequently, by uniting the system identification and simulation components of the modeling procedure into an integrated method, we can develop a cyclical flow from modeling through experiments through updates to the global biological knowledge base. Such a flow is designed specifically to respond to the challenges of designing and interpreting high-throughput experiments, which can in the future evolve in concert with modeling and information management.

Compared to previous models such as Boolean network model [35,23,36] and difference/differential equation [17,18], the proposed model (1) has the following characteristics. Firstly, gene expression profiles are the observation variables rather than the internal state variables. Secondly, from a biological angle, the model (1) can capture the fact that genes may be regulated by internal regulatory factor [37]. Thirdly, the model (1) takes the control portion of state-space model into consideration. However, the proposed approach does have some shortcomings, for example, the inherent linearity which can only capture the primary linear components of a biological system which may be nonlinear, and the ignorance to time delays in a biological system resulting, for example, from the time necessary for transcription, translation, and diffusion. These shortcomings will be address in the future work.

## Methods

### The data flow of our approach

**Figure 8:**
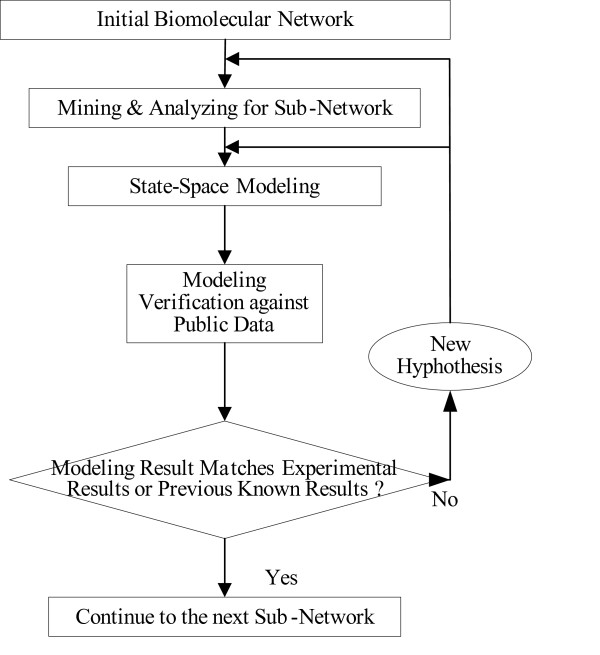
Outline of Mining and Modeling of Biomolecular Network.

The dataflow of our method is illustrated in Figure 8. A novel scale-free network clustering approach is applied to the biomolecular network to obtain various sub-networks. Then hypothetical state-space base model is generated for the sub-networks and simulate them to predict their dynamic biological behavior. The modeling results are verified against high-throughput data (microarrays and/or genetic screens) for both the natural system and perturbations. If computational results do not match experimental or previously published results, then a new hypothesis is generated and fed back to the data mining and analyzing step to refine the biomolecular network for the next iteration as a better convergence between continuous modeling and experiments evolves. Notably, the dynamic modeling component of this method depends on the automated network structure generation of the first component and the sub-network clustering, which are both essential to make the solution tractable. The details of steps are described in details in the subsequent sections

### Analyzing biomolecular network to identify sub-networks

The interpretation of large-scale protein network data depends on our ability to identify significant sub-structures (communities) in the data, a computationally intensive task. Many algorithms for detecting community structure in networks have been proposed. They can be roughly classified into two categories, divisive and agglomerative. The divisive approach takes the route of recursive removal of vertices (or edges) until the network is separated into its components or communities, whereas the agglomerative approach starts with isolated individual vertices and joins together small communities. One important algorithm is proposed by Girvan and Newman (the GN algorithm) [38]. The GN algorithm is based on the concept of betweenness, a quantitative measure of the number of shortest paths passing through a given vertex (or edge). The GN algorithm detects communities in a network by recursively removing these high betweenness vertices (or edges). It has produced good results and is well adopted by different authors in studying various networks [1]. However, it has a major disadvantage which is its computational cost. For sparse networks with n vertices, the GN algorithm is of O(*n*^3^) time. Various alternative algorithms have been proposed [39-42], attempting to improve either the quality of the community structure or the computational efficiency. As discussed by Holme et al [43], edge-betweenness uses properties calculated from the whole graph, allowing information from non-local features to be used in the clustering. Edge-betweenness algorithm does not scale well to larger graphs, this method is currently most appropriate for studies focused on specific areas of the proteome.

The goal of our work here is to address a slightly different question about the community structure in a biomolecular network, i.e., what is the community to which a given protein (or proteins) belongs? We are motivated by two main factors. Firstly, due to the complexity and modularity of biological networks, it is more feasible computationally to study a community containing a small number of proteins of interest. Secondly, sometimes the whole community structure of the network may not be our primary concern. Rather, we may be more interested in finding the community which contains a protein (or proteins) of interest. Our aim is to discover relatively small sub-networks such that proteins inside the sub-network interact significantly and, meanwhile, they are not strongly influenced by proteins outside the sub-network. Sub-networks are constructed starting with a seed consisting of one or more proteins believed to be participated in a viable sub-network. Functionalities and regulatory relationships among seed proteins may be partially known or they may simply be of interest. Given the seed, we iteratively adjoin new proteins following an adapted definition of a community in a network. The sub-networks built from our models may provide valuable theoretical guidance to experiment.

### The algorithm SNBuilder (sub-network builder)

We intuitively model the protein-protein interaction network as an undirected graph, where vertices represent proteins and edges represent interactions between pairs of proteins. An undirected graph, *G *= (*V*, *E*), is comprised of two sets, vertices *V *and edges *E*. An edge *e *is defined as a pair of vertices (*u, v*) denoting the direct connection between vertices *u *and *v*. The graphs we use in this paper are undirected, unweighted, and simple – meaning no self-loops or parallel edges.

For a subgraph *G' *⊂ *G *and a vertex *i *belonging to *G'*, we define the in-community degree for vertex *i*, kiin
 MathType@MTEF@5@5@+=feaafiart1ev1aaatCvAUfKttLearuWrP9MDH5MBPbIqV92AaeXatLxBI9gBaebbnrfifHhDYfgasaacH8akY=wiFfYdH8Gipec8Eeeu0xXdbba9frFj0=OqFfea0dXdd9vqai=hGuQ8kuc9pgc9s8qqaq=dirpe0xb9q8qiLsFr0=vr0=vr0dc8meaabaqaciaacaGaaeqabaqabeGadaaakeaacqWGRbWAdaqhaaWcbaGaemyAaKgabaGaemyAaKMaemOBa4gaaaaa@3253@(*G'*), to be the number of edges connecting vertex *i *to other vertices belonging to *G' *and the out-community degree, kiout
 MathType@MTEF@5@5@+=feaafiart1ev1aaatCvAUfKttLearuWrP9MDH5MBPbIqV92AaeXatLxBI9gBaebbnrfifHhDYfgasaacH8akY=wiFfYdH8Gipec8Eeeu0xXdbba9frFj0=OqFfea0dXdd9vqai=hGuQ8kuc9pgc9s8qqaq=dirpe0xb9q8qiLsFr0=vr0=vr0dc8meaabaqaciaacaGaaeqabaqabeGadaaakeaacqWGRbWAdaqhaaWcbaGaemyAaKgabaGaem4Ba8MaemyDauNaemiDaqhaaaaa@33DE@(*G'*), to be the number of edges connecting vertex *i *to other vertices that are in *G *but do not belong to *G'*.

In our algorithm, we adopt the quantitative definitions of community defined by Radicchi and colleagues [33], i.e. the subgraph *G' *is a community in a strong sense if kiin
 MathType@MTEF@5@5@+=feaafiart1ev1aaatCvAUfKttLearuWrP9MDH5MBPbIqV92AaeXatLxBI9gBaebbnrfifHhDYfgasaacH8akY=wiFfYdH8Gipec8Eeeu0xXdbba9frFj0=OqFfea0dXdd9vqai=hGuQ8kuc9pgc9s8qqaq=dirpe0xb9q8qiLsFr0=vr0=vr0dc8meaabaqaciaacaGaaeqabaqabeGadaaakeaacqWGRbWAdaqhaaWcbaGaemyAaKgabaGaemyAaKMaemOBa4gaaaaa@3253@(*G'*) > kiout
 MathType@MTEF@5@5@+=feaafiart1ev1aaatCvAUfKttLearuWrP9MDH5MBPbIqV92AaeXatLxBI9gBaebbnrfifHhDYfgasaacH8akY=wiFfYdH8Gipec8Eeeu0xXdbba9frFj0=OqFfea0dXdd9vqai=hGuQ8kuc9pgc9s8qqaq=dirpe0xb9q8qiLsFr0=vr0=vr0dc8meaabaqaciaacaGaaeqabaqabeGadaaakeaacqWGRbWAdaqhaaWcbaGaemyAaKgabaGaem4Ba8MaemyDauNaemiDaqhaaaaa@33DE@(*G'*) for each vertex *i *in *G' *and in a weak sense if the sum of all degrees within *G' *is greater than the sum of all degrees from *G' *to the rest of the graph.

The algorithm shown in Table 2, called *SNBuilder*, accepts the seed protein *s*, gets the neighbors of *s*, finds the core of the community to build, and expands the core to find the eventual community. The two major components of *SNBuilder *are *FindCore *and *ExpandCore*. In fact, *FindCore *(line 8 to line 14) performs a naïve search for maximum clique from the neighborhood of the seed protein by recursively removing vertices with the lowest in-community degree until either 1) all vertices in the core set have the same in-community degree (*K*_*min *_= *K*_*max*_, i.e. the resulting subgraph is a clique); or 2) all vertices except the seed have the same in-community degree (a star-like structure).

**Table 2:** Names of genes and proteins

1:	*G*(*V, E*) is the input graph with vertex set *V *and edge set *E*.
2:	*s *is the seed vertex; *f *is the affinity threshold; *d *is the distance threshold.
3:	*N *← {Adjacency list of *s*} ⋃ {*s*}
4:	*C *← FindCore(*N*)
5:	*C' *← ExpandCore(*C, f, d*)
6:	**return ***C'*
7:	FindCore(*N*)
8:	**for each ***v *∈ *N*
9:	calculate kvin MathType@MTEF@5@5@+=feaafiart1ev1aaatCvAUfKttLearuWrP9MDH5MBPbIqV92AaeXatLxBI9gBaebbnrfifHhDYfgasaacH8akY=wiFfYdH8Gipec8Eeeu0xXdbba9frFj0=OqFfea0dXdd9vqai=hGuQ8kuc9pgc9s8qqaq=dirpe0xb9q8qiLsFr0=vr0=vr0dc8meaabaqaciaacaGaaeqabaqabeGadaaakeaacqWGRbWAdaqhaaWcbaGaemODayhabaGaemyAaKMaemOBa4gaaaaa@326D@(*N*)
10:	**end for**
11:	*K*_*min *_← min {kvin MathType@MTEF@5@5@+=feaafiart1ev1aaatCvAUfKttLearuWrP9MDH5MBPbIqV92AaeXatLxBI9gBaebbnrfifHhDYfgasaacH8akY=wiFfYdH8Gipec8Eeeu0xXdbba9frFj0=OqFfea0dXdd9vqai=hGuQ8kuc9pgc9s8qqaq=dirpe0xb9q8qiLsFr0=vr0=vr0dc8meaabaqaciaacaGaaeqabaqabeGadaaakeaacqWGRbWAdaqhaaWcbaGaemODayhabaGaemyAaKMaemOBa4gaaaaa@326D@(*N*), *v *∈ *N*}
12:	*K*_*max *_← max {kvin MathType@MTEF@5@5@+=feaafiart1ev1aaatCvAUfKttLearuWrP9MDH5MBPbIqV92AaeXatLxBI9gBaebbnrfifHhDYfgasaacH8akY=wiFfYdH8Gipec8Eeeu0xXdbba9frFj0=OqFfea0dXdd9vqai=hGuQ8kuc9pgc9s8qqaq=dirpe0xb9q8qiLsFr0=vr0=vr0dc8meaabaqaciaacaGaaeqabaqabeGadaaakeaacqWGRbWAdaqhaaWcbaGaemODayhabaGaemyAaKMaemOBa4gaaaaa@326D@(*N*), *v *∈ *N*}
13:	**if ***K*_*min *_= *K*_*max *_or (kiin(N)=kjin(N), ∀i,j∈N,i,j≠s,i≠j MathType@MTEF@5@5@+=feaafiart1ev1aaatCvAUfKttLearuWrP9MDH5MBPbIqV92AaeXatLxBI9gBaebbnrfifHhDYfgasaacH8akY=wiFfYdH8Gipec8Eeeu0xXdbba9frFj0=OqFfea0dXdd9vqai=hGuQ8kuc9pgc9s8qqaq=dirpe0xb9q8qiLsFr0=vr0=vr0dc8meaabaqaciaacaGaaeqabaqabeGadaaakeaacqWGRbWAdaqhaaWcbaGaemyAaKgabaGaemyAaKMaemOBa4gaaOGaeiikaGIaemOta4KaeiykaKIaeyypa0Jaem4AaS2aa0baaSqaaiabdQgaQbqaaiabdMgaPjabd6gaUbaakiabcIcaOiabd6eaojabcMcaPiabcYcaSiabbccaGiabgcGiIiabdMgaPjabcYcaSiabdQgaQjabgIGiolabd6eaojabcYcaSiabdMgaPjabcYcaSiabdQgaQjabgcMi5kabdohaZjabcYcaSiabdMgaPjabgcMi5kabdQgaQbaa@5489@) **then return ***N*
14:	**else return **FindCore(*N *- {*v*}, kvin MathType@MTEF@5@5@+=feaafiart1ev1aaatCvAUfKttLearuWrP9MDH5MBPbIqV92AaeXatLxBI9gBaebbnrfifHhDYfgasaacH8akY=wiFfYdH8Gipec8Eeeu0xXdbba9frFj0=OqFfea0dXdd9vqai=hGuQ8kuc9pgc9s8qqaq=dirpe0xb9q8qiLsFr0=vr0=vr0dc8meaabaqaciaacaGaaeqabaqabeGadaaakeaacqWGRbWAdaqhaaWcbaGaemODayhabaGaemyAaKMaemOBa4gaaaaa@326D@(*N*) = *K*_*min*_)
15:	ExpandCore(*C, f, d*)
16:	D→∪(v,w)∈E,v∈C,w∉C{v,w} MathType@MTEF@5@5@+=feaafiart1ev1aaatCvAUfKttLearuWrP9MDH5MBPbIqV92AaeXatLxBI9gBaebbnrfifHhDYfgasaacH8akY=wiFfYdH8Gipec8Eeeu0xXdbba9frFj0=OqFfea0dXdd9vqai=hGuQ8kuc9pgc9s8qqaq=dirpe0xb9q8qiLsFr0=vr0=vr0dc8meaabaqaciaacaGaaeqabaqabeGadaaakeaacqWGebarcqGHsgIRdaWfqaqaaiabgQIiidWcbaGaeiikaGIaemODayNaeiilaWIaem4DaCNaeiykaKIaeyicI4SaemyrauKaeiilaWIaemODayNaeyicI4Saem4qamKaeiilaWIaem4DaCNaeyycI8Saem4qameabeaakiabcUha7jabdAha2jabcYcaSiabdEha3jabc2ha9baa@4A42@
17:	*C' *← *C*
18:	**for each ***t *∈ *D*, *t *∉ *C*, and distance(*t*, *s*) <= *d*
19:	calculate ktin MathType@MTEF@5@5@+=feaafiart1ev1aaatCvAUfKttLearuWrP9MDH5MBPbIqV92AaeXatLxBI9gBaebbnrfifHhDYfgasaacH8akY=wiFfYdH8Gipec8Eeeu0xXdbba9frFj0=OqFfea0dXdd9vqai=hGuQ8kuc9pgc9s8qqaq=dirpe0xb9q8qiLsFr0=vr0=vr0dc8meaabaqaciaacaGaaeqabaqabeGadaaakeaacqWGRbWAdaqhaaWcbaGaemiDaqhabaGaemyAaKMaemOBa4gaaaaa@3269@(*D*)
20:	calculate ktout MathType@MTEF@5@5@+=feaafiart1ev1aaatCvAUfKttLearuWrP9MDH5MBPbIqV92AaeXatLxBI9gBaebbnrfifHhDYfgasaacH8akY=wiFfYdH8Gipec8Eeeu0xXdbba9frFj0=OqFfea0dXdd9vqai=hGuQ8kuc9pgc9s8qqaq=dirpe0xb9q8qiLsFr0=vr0=vr0dc8meaabaqaciaacaGaaeqabaqabeGadaaakeaacqWGRbWAdaqhaaWcbaGaemiDaqhabaGaem4Ba8MaemyDauNaemiDaqhaaaaa@33F4@(*D*)
21:	**if **ktin MathType@MTEF@5@5@+=feaafiart1ev1aaatCvAUfKttLearuWrP9MDH5MBPbIqV92AaeXatLxBI9gBaebbnrfifHhDYfgasaacH8akY=wiFfYdH8Gipec8Eeeu0xXdbba9frFj0=OqFfea0dXdd9vqai=hGuQ8kuc9pgc9s8qqaq=dirpe0xb9q8qiLsFr0=vr0=vr0dc8meaabaqaciaacaGaaeqabaqabeGadaaakeaacqWGRbWAdaqhaaWcbaGaemiDaqhabaGaemyAaKMaemOBa4gaaaaa@3269@ (*D*) > ktout MathType@MTEF@5@5@+=feaafiart1ev1aaatCvAUfKttLearuWrP9MDH5MBPbIqV92AaeXatLxBI9gBaebbnrfifHhDYfgasaacH8akY=wiFfYdH8Gipec8Eeeu0xXdbba9frFj0=OqFfea0dXdd9vqai=hGuQ8kuc9pgc9s8qqaq=dirpe0xb9q8qiLsFr0=vr0=vr0dc8meaabaqaciaacaGaaeqabaqabeGadaaakeaacqWGRbWAdaqhaaWcbaGaemiDaqhabaGaem4Ba8MaemyDauNaemiDaqhaaaaa@33F4@ (*D*) **or **ktin MathType@MTEF@5@5@+=feaafiart1ev1aaatCvAUfKttLearuWrP9MDH5MBPbIqV92AaeXatLxBI9gBaebbnrfifHhDYfgasaacH8akY=wiFfYdH8Gipec8Eeeu0xXdbba9frFj0=OqFfea0dXdd9vqai=hGuQ8kuc9pgc9s8qqaq=dirpe0xb9q8qiLsFr0=vr0=vr0dc8meaabaqaciaacaGaaeqabaqabeGadaaakeaacqWGRbWAdaqhaaWcbaGaemiDaqhabaGaemyAaKMaemOBa4gaaaaa@3269@ (*D*)/|*D*| > *f ***then ***C' *← *C' *∪ {*t*}
22:	**end for**
23:	**if ***C' *= *C ***then return ***C*
24:	**else return **ExpandCore(*C', f, d*)

The algorithm performs a breadth first expansion in the core expanding step. It first builds a candidate set containing the core and all vertices adjacent to each vertex in the core (line 16). A candidate vertex will then be added to the core if it meets one of the following conditions (line 21): 1) its in-community degree is greater than its out-community degree, i.e. the quantitative definition of community in a strong sense (ktin
 MathType@MTEF@5@5@+=feaafiart1ev1aaatCvAUfKttLearuWrP9MDH5MBPbIqV92AaeXatLxBI9gBaebbnrfifHhDYfgasaacH8akY=wiFfYdH8Gipec8Eeeu0xXdbba9frFj0=OqFfea0dXdd9vqai=hGuQ8kuc9pgc9s8qqaq=dirpe0xb9q8qiLsFr0=vr0=vr0dc8meaabaqaciaacaGaaeqabaqabeGadaaakeaacqWGRbWAdaqhaaWcbaGaemiDaqhabaGaemyAaKMaemOBa4gaaaaa@3269@ > ktout
 MathType@MTEF@5@5@+=feaafiart1ev1aaatCvAUfKttLearuWrP9MDH5MBPbIqV92AaeXatLxBI9gBaebbnrfifHhDYfgasaacH8akY=wiFfYdH8Gipec8Eeeu0xXdbba9frFj0=OqFfea0dXdd9vqai=hGuQ8kuc9pgc9s8qqaq=dirpe0xb9q8qiLsFr0=vr0=vr0dc8meaabaqaciaacaGaaeqabaqabeGadaaakeaacqWGRbWAdaqhaaWcbaGaemiDaqhabaGaem4Ba8MaemyDauNaemiDaqhaaaaa@33F4@); 2) its affinity coefficient is greater than or equals to the affinity threshold *f*.

We define the affinity coefficient of a vertex to a network as the fraction of its in-community degree over the size of the network excluding the vertex itself (ktin
 MathType@MTEF@5@5@+=feaafiart1ev1aaatCvAUfKttLearuWrP9MDH5MBPbIqV92AaeXatLxBI9gBaebbnrfifHhDYfgasaacH8akY=wiFfYdH8Gipec8Eeeu0xXdbba9frFj0=OqFfea0dXdd9vqai=hGuQ8kuc9pgc9s8qqaq=dirpe0xb9q8qiLsFr0=vr0=vr0dc8meaabaqaciaacaGaaeqabaqabeGadaaakeaacqWGRbWAdaqhaaWcbaGaemiDaqhabaGaemyAaKMaemOBa4gaaaaa@3269@ (*D*)/(|*D*|-1)). We introduce the affinity coefficient and the affinity threshold *f *to provide a degree of relaxation when expanding the core, because it is too strict requiring every expanding vertex to be a strong sense community member. Even though a candidate vertex may not have an in-community degree larger than out-community degree, it may connect to all (or even most of) other members of the network, indicating a strong tie between the candidate vertex and the network. We use an affinity threshold *f *of 1 in our implementation, meaning that in order to be eligible to add to the core set, the candidate vertex has to connect to all other vertices in the core set. However, *f *may be relaxed to be less than 1 if necessary or so desired.

In addition, a distance parameter (*d*) is provided to restrict how far away a candidate vertex to the seed can be considered eligible for expansion. Quite often, a given seed may not always situate in the center of the resulting sub-network. The distance parameter serves as the shortest path threshold to ensure that all members of the obtained sub-network will be within specified proximity to the seed. A large enough value of *d*, such as one that is larger than the longest path from the seed to all other vertices in the network, will virtually lift this distance restriction.

The *FindCore *is a heuristic search for a maximum complete subgraph in the neighborhood *N *of seed *s*. Let *K *be the size of *N*, then the worst-case running time of *FindCore *is *O*(*K*^2^). The *ExpandCore *part costs in worst-case approximately |*V*| + |*E*| + overhead. |*V*| accounts for the expanding of the core, at most all vertices in *V*, minus what are already in the core, would be included. |*E*| accounts for calculating the in- and out-degrees for the candidate vertices that are not in the core but in the neighborhood of the core. The overhead is caused by recalculating the in- and out-degrees of neighboring vertices every time the *FindCore *is recursively called. The number of these vertices is dependent on the size of the community we are building and the connectivity of the community to the rest of the network, but not the overall size of the network. For biological networks, the graphs we deal with are mostly sparse and small world, therefore, the running time of our algorithm is close to linear.

### Evaluation of SNBuilder

Because there is no alternative approach to our method, we decide to compare the performance of our algorithm to the work on predicting protein complex membership by Asthana and colleagues [32]. Asthana and colleagues reported results of queries with ***four ***complexes using probabilistic network reliability (we will refer their work as PNR method in the following discussion). ***Four communities ***are identified by SNBuilder using one protein as seed from each of the query complexes used by the PNR method. (Because of the space limitation, we only discuss the comparison study of 1 out of the 4 complexes). The seed protein is selected randomly from the "core" protein set. The figures for visualizing the identified communities are created using Pajek [44]. The community figures are extracted from the network we build using the above mentioned data set with out-of-community connections omitted. The proteins in each community are annotated with a brief description obtained from the MIPS complex catalogue database. As a comparison, we use Complexpander, an implementation of the PNR method [32] to predict co-complex using the core protein set that contains the same seed protein used by SNBuilder. For all our queries when using Complexpander, we select the option to use the MIPS complex catalogue database. We record the ranking of the members in our identified communities that also appear in the co-complex candidate list predicted by Comlexpander.

**Figure 9:**
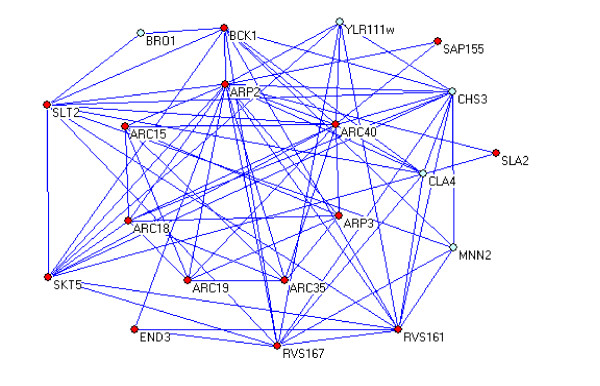
The ARP2/ARP3 community.

In our example, we use ARP3 as seed to identify the community (Figure 9). ARP2/ARP3 complex acts as multi-functional organizer of actin filaments. The assembly and maintenance of many actin-based cellular structures likely depend on functioning ARP2/ARP3 complex [45]. The identified community contains all 7 proteins of the ARP2/ARP3 complex listed in MIPS (Table 3). Not including the seed (ARP3), these proteins represent the top 6 ranked proteins predicted by Complexpander. As indicated in Table 3, there are 14 members belonging to the same functional category of budding, cell polarity, and filament formation, according to MIPS. For more experimental comparison results, please see the supplementary data file 1

**Table 3:** The ARP2/ARP3 community

Protein	*Alias*	*Description*	*Rank*
YLR111w	YLR111w	hypothetical protein	
YIL062c	ARC15*†	subunit of the Arp2/3 complex	1
YLR370c	ARC18*	subunit of the Arp2/3 complex	4
YKL013c	ARC19*†	subunit of the Arp2/3 complex	3
YNR035c	ARC35*	subunit of the Arp2/3 complex	5
YBR234c	ARC40*†	Arp2/3 protein complex subunit, 40 kilodalton	6
YDL029w	ARP2*†	actin-like protein	2
YJR065c	ARP3*	actin related protein	
YJL095w	BCK1†	ser/thr protein kinase of the MEKK family	
YPL084w	BRO1	required for normal response to nutrient limitation	
YBR023c	CHS3†	chitin synthase III	
YNL298w	CLA4†	ser/thr protein kinase	
YNL084c	END3†	required for endocytosis and cytoskeletal organization	
YBR015c	MNN2	type II membrane protein	
YCR009c	RVS161†	protein involved in cell polarity development	
YDR388w	RVS167†	reduced viability upon starvation protein	
YFR040w	SAP155†	Sit4p-associated protein	
YBL061c	SKT5†	protoplast regeneration and killer toxin resistance protein	
YNL243w	SLA2†	cytoskeleton assembly control protein	
YHR030c	SLT2†	ser/thr protein kinase of MAP kinase family	

*Proteins belonging to ARP2/3 complex listed in MIPS.

†Proteins listed in the functional category of budding, cell polarity, and filament formation by MIPS.

### State-pace based simulation of the biomolecular network

**Figure 10:**
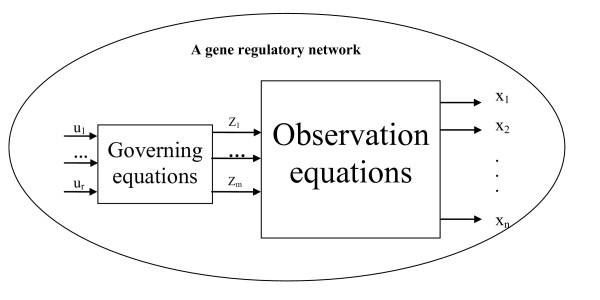
A state-space model for gene regulatory networks, where *x*_*i*_(*i *= 1,⋯,*n*) are the observation variables, *z*_*i*_(*i *= 1,⋯*p*) are the state variables, and *u*_*i*_(*i *= 1,⋯*r*) are the control variables.

The state-space approach is one of the most powerful methods to modeling a dynamic system and has been widely employed for engineering control systems [46]. A state-space model consists of internal variables, external input variables, and output (observation) variables. In a state-space model, the observation variables typically depend on the internal variables, while the change in the internal variables is completely determined by the current internal variables plus any external inputs. In the existing models such as Boolean network, differential and difference models, genes are viewed as the internal state variables as well as observation variables of a biomolecular network, and their expression levels are the values of both the internal state variables and the observation variables. This viewpoint has suffered from the underestimation of the model parameters as pointed out previously. Actually, not all genes (their products, proteins) directly regulate gene expressions in a network since only a part of genes are translated into regulatory factors (proteins) which regulate gene expression while others are translated into structure proteins which do not participate gene regulation [37,47,48]. Recently we have propose a state-space model for gene regulatory networks [19], in which genes are viewed as the observation variables and gene expression dynamics is governed by a group of the internal variables as shown in Figure 10. Further we have extended this model to take time-delayed regulatory relationships [49,50]. However, the previous model [19] does not take the control portion of the system into consideration, and instead just describes the influence of internal states on gene expression level and assumes that the internal states evolve autonomously. Actually, expression levels of all genes affect the internal states in turn.

### Mathematic model

The following state-space model is proposed to describe a gene regulatory network

{z(t+1)=A⋅z(t)+Bu(t)+n1(t)x(t)=C⋅z(t)+n2(t)
 MathType@MTEF@5@5@+=feaafiart1ev1aaatCvAUfKttLearuWrP9MDH5MBPbIqV92AaeXatLxBI9gBaebbnrfifHhDYfgasaacH8akY=wiFfYdH8Gipec8Eeeu0xXdbba9frFj0=OqFfea0dXdd9vqai=hGuQ8kuc9pgc9s8qqaq=dirpe0xb9q8qiLsFr0=vr0=vr0dc8meaabaqaciaacaGaaeqabaqabeGadaaakeaadaGabaabaeqabaacbmGae8NEaONaeiikaGIaemiDaqNaey4kaSIaeGymaeJaeiykaKIaeyypa0Jae8xqaeKaeyyXICTae8NEaONaeiikaGIaemiDaqNaeiykaKIaey4kaSIae8NqaiKae8xDauNaeiikaGIaemiDaqNaeiykaKIaey4kaSIae8NBa42aaSbaaSqaaiabigdaXaqabaGccqGGOaakcqWG0baDcqGGPaqkaeaacqWF4baEcqGGOaakcqWG0baDcqGGPaqkcqGH9aqpcqWFdbWqcqGHflY1cqWF6bGEcqGGOaakcqWG0baDcqGGPaqkcqGHRaWkcqWFUbGBdaWgaaWcbaGaeGOmaidabeaakiabcIcaOiabdsha0jabcMcaPaaacaGL7baaaaa@5E60@

The meaning of the variables follows as: in terms of linear system theory [45], equations (1) are called the state-space model of a dynamic system. The vector ***x***(*t*) = [*x*_1_(*t*) ⋯ *x*_*n*_(*t*)]^*T *^consists of the observation variables of the system and *x*_*i*_(*t*)(*i *= 1,⋯,*n*) represents the expression level of gene *i *at time point *t*, where *n *is the number of genes in the network. The vector ***z***(*t*) = [*z*_1_(*t*) ⋯ *z*_*p*_(*t*)]^*T *^consists of the internal state variables of the system and *z*_*i*_(*t*)(*i *= 1,⋯,*p*) represents the expression value of internal element (variable) *i *at time point *t *which directly regulates gene expression, where *p *is the number of the internal state variables. The vector ***u***(*t*) = [*u*_1_(*t*) ⋯ *u*_*r*_(*t*)]^*T *^represents the external input (control variable) of the internal state governing equation. The matrix ***A ***= [*a*_*ij*_]_*p*×*p *_is the time translation matrix of the internal state variables or the state transition matrix. It provides key information on the influences of the internal variables on each other. The matrix *B *= [*b*_*ik*_]_*p*×*r *_is the control matrix. The entries of the matrix reflect the strength of a control variable to an internal variable. The matrix ***C ***= [*c*_*ik*_]_*n*×*p *_is the observation matrix which transfers the information from the internal state variables to the observation variables. The entries of the matrix encode information on the influences of the internal regulatory elements on the genes. Finally, the vectors ***n***_1_(*t*) and ***n***_2_(*t*) stand for system noise and observation noise. In model (1) the upper equation is called the internal state governing equation while the lower one is called the observation equation.

### Parameter estimation

Let ***X ***be the gene expression data matrix with *n *rows and *m *columns, where *n *and *m *are the numbers of the genes and the measuring time points, respectively. The constructing of model (1) using microarray gene expression data ***X ***may be divided into three phases. Phase one identifies the internal state variables and their expression matrix, and estimates the elements of observation matrix ***C***; Phase two defines the control internal variables; and Phase three estimates the elements of matrices ***A ***and ***B***.

#### Internal variables and estimation of observation matrix

The internal states are latent variables in gene regulatory networks. They could be any unobserved molecules in cell which participate the process of gene regulation. From the biological viewpoint, it is reasonable to assume that the latent variables are some regulatory factors (protein) or missed genes. Many statistical methods [51] have been developed to find the expression of latent variables from the observation data. In this study, the maximum-likelihood algorithm for probabilistic principal component analysis PPCA [30] is employed to extract the internal variables from the observation data (time-course gene expression data). Using the PPCA model, it follows that

***X ***= ***C***·***Z ***+ *N*

where **X **is the *n*×*m *observation data matrix, each column of which is viewed as an observation sample; **C **is the *n*×*p *transformation matrix, and ***z ***represents the expression profile of an internal state, and *N *is the *n*×*m *noise matrix consisting by *m *n-dimensional observation noise vectors. Assume that the sample mean is shifted to zero. The log-likelihood of PPCA model [30] is expressed by

L=−m2{n(ln⁡2π)+log⁡|D|+tr(D−1S)}
 MathType@MTEF@5@5@+=feaafiart1ev1aaatCvAUfKttLearuWrP9MDH5MBPbIqV92AaeXatLxBI9gBaebbnrfifHhDYfgasaacH8akY=wiFfYdH8Gipec8Eeeu0xXdbba9frFj0=OqFfea0dXdd9vqai=hGuQ8kuc9pgc9s8qqaq=dirpe0xb9q8qiLsFr0=vr0=vr0dc8meaabaqaciaacaGaaeqabaqabeGadaaakeaacqWGmbatcqGH9aqpcqGHsisldaWcaaqaaiabd2gaTbqaaiabikdaYaaadaGabaqaaiabd6gaUbGaay5EaaWaaiGaaeaacqGGOaakcyGGSbaBcqGGUbGBcqaIYaGmiiGacqWFapaCcqGGPaqkcqGHRaWkcyGGSbaBcqGGVbWBcqGGNbWzdaabdaqaaiabdseaebGaay5bSlaawIa7aiabgUcaRiabdsha0jabdkhaYjabcIcaOiabdseaenaaCaaaleqabaGaeyOeI0IaeGymaedaaOGaem4uamLaeiykaKcacaGL9baaaaa@4FE8@

where *D *= *CC*^*T *^+ *σ*^2^*I *and *σ*^2^is the variance of the observation noise, and ***S ***= X * *X'*/*m*. For the given number of internal variables, *p*, the global maximum log-likelihood of the PPCA model is calculated by

Lp=−m2{∑j=1plog⁡(λj)+(n−p)*log⁡(∑j=p+1nλj/(n−p))+n(log⁡(2π)+1)}
 MathType@MTEF@5@5@+=feaafiart1ev1aaatCvAUfKttLearuWrP9MDH5MBPbIqV92AaeXatLxBI9gBaebbnrfifHhDYfgasaacH8akY=wiFfYdH8Gipec8Eeeu0xXdbba9frFj0=OqFfea0dXdd9vqai=hGuQ8kuc9pgc9s8qqaq=dirpe0xb9q8qiLsFr0=vr0=vr0dc8meaabaqaciaacaGaaeqabaqabeGadaaakeaacqWGmbatdaWgaaWcbaGaemiCaahabeaakiabg2da9iabgkHiTmaalaaabaGaemyBa0gabaGaeGOmaidaamaaceaabaWaaabCaeaacyGGSbaBcqGGVbWBcqGGNbWzcqGGOaakiiGacqWF7oaBdaWgaaWcbaGaemOAaOgabeaakiabcMcaPiabgUcaRiabcIcaOiabd6gaUjabgkHiTiabdchaWjabcMcaPiabcQcaQaWcbaGaemOAaOMaeyypa0JaeGymaedabaGaemiCaahaniabggHiLdaakiaawUhaamaaciaabaGagiiBaWMaei4Ba8Maei4zaC2aaeWaaeaadaWcgaqaamaaqahabaGae83UdW2aaSbaaSqaaiabdQgaQbqabaaabaGaemOAaOMaeyypa0JaemiCaaNaey4kaSIaeGymaedabaGaemOBa4ganiabggHiLdaakeaacqGGOaakcqWGUbGBcqGHsislcqWGWbaCcqGGPaqkaaaacaGLOaGaayzkaaGaey4kaSIaemOBa4MaeiikaGIagiiBaWMaei4Ba8Maei4zaCMaeiikaGIaeGOmaiJae8hWdaNaeiykaKIaey4kaSIaeGymaeJaeiykaKcacaGL9baaaaa@7337@

when

*C *= ***U***_*p*_

where *λ*_*j *_(*j *= 1,⋯,*p*) are the first *p *largest eigenvalues of the sample variance matrix **S**, **U**_*p *_is a *n*×*p *matrix, each column of which is a corresponding eigenvector of **S**.

From Equation (4), the values of the maximum log-likelihood for the PPCA model increase with the increased numbers of internal state variables, *p*. The redundant internal state variables may result in a complicated model. Since the PPCA has a solid probabilistic foundation, some statistics-based criteria such as BIC and AIC can be used to determine the number of internal state variables [52,53]. As the number of genes is small in a sub-network, in this paper, the AIC is adopted. For each model, the AIC is define

*AIC*(*p*) = 2·*L*_*p *_- 2·*v*_*p*_

where *v*_*p *_(=*np*+1) is the number of parameters (elements of matrix C) in the PPCA model. Since the terms *nm*(log(2*π*)+1)/2 in Equation (6) is a constant for a given dataset, the calculation of AIC can be simplified as

AIC(p)=−m{∑j=1plog⁡(λj)+(n−p)*log⁡(∑j=k+1nλj/(n−p))}−2⋅(np+1)
 MathType@MTEF@5@5@+=feaafiart1ev1aaatCvAUfKttLearuWrP9MDH5MBPbIqV92AaeXatLxBI9gBaebbnrfifHhDYfgasaacH8akY=wiFfYdH8Gipec8Eeeu0xXdbba9frFj0=OqFfea0dXdd9vqai=hGuQ8kuc9pgc9s8qqaq=dirpe0xb9q8qiLsFr0=vr0=vr0dc8meaabaqaciaacaGaaeqabaqabeGadaaakeaacqWGbbqqcqWGjbqscqWGdbWqcqGGOaakcqWGWbaCcqGGPaqkcqGH9aqpcqGHsislcqWGTbqBdaGabaqaamaaqahabaGagiiBaWMaei4Ba8Maei4zaCMaeiikaGccciGae83UdW2aaSbaaSqaaiabdQgaQbqabaGccqGGPaqkcqGHRaWkcqGGOaakcqWGUbGBcqGHsislcqWGWbaCcqGGPaqkcqGGQaGkaSqaaiabdQgaQjabg2da9iabigdaXaqaaiabdchaWbqdcqGHris5aaGccaGL7baadaGacaqaaiGbcYgaSjabc+gaVjabcEgaNnaabmaabaWaaSGbaeaadaaeWbqaaiab=T7aSnaaBaaaleaacqWGQbGAaeqaaaqaaiabdQgaQjabg2da9iabdUgaRjabgUcaRiabigdaXaqaaiabd6gaUbqdcqGHris5aaGcbaGaeiikaGIaemOBa4MaeyOeI0IaemiCaaNaeiykaKcaaaGaayjkaiaawMcaaaGaayzFaaGaeyOeI0IaeGOmaiJaeyyXICTaeiikaGIaemOBa4MaemiCaaNaey4kaSIaeGymaeJaeiykaKcaaa@71F1@

By this definition, the model with the largest AIC is chosen. After the transformation matrix **C **is determined, the expression profiles of internal variables accumulated in matrix *Z *can be calculated by formula

*Z *= *C*^+^**X**

#### Control variables, network structure, and control matrix

In state-space model (1), the control variables together with current internal states determine the next internal states. From the viewpoint of biology, the overall expression level of all genes in the network affects the internal (hide) variables [46,47,54]. In this study, we take *u*(*t*) = *x*(*t*) as the input of the internal state equation. Therefore, from the model (1), it follows that

*x*(*t *+ 1) = *CAz*(*t*) + *CBx*(*t*)

This equation quantitatively describes the regulatory relationships among genes through the matrix *CB*. On the other hand, using the algorithm SNBuilder, the subnetwork can be presented by a graph as shown in Figure 1. In this paper, the adjacent matrix of such a graph is called the structure matrix of the network as it qualitatively describes the regulatory relationships among genes, denoted by S. Therefore the structure of matrix *CB *should be the same as that of matrix *S*. That is, the (i, j)-th element of *CB *is nonzero (or zero) if the (i, j)-th element of *S *is nonzero (or zero).

It is nontrivial to find a control matrix *B *such that the structure of matrix *CB *is the same as that of matrix *S*. Considering that in reality the weak connections among genes may be ignored in the structure of the network, we reformulate the problem as follows: find a matrix *B *such the squared sum over the elements of *CB *corresponding to non-zeros in S is much larger than that over other elements.

#### Estimates of state transition matrix

With the calculated control matrix *B *and the profiles of internal variables *Z*, one can estimate the parameters of the state transition matrix in the internal state governing equation:

***z***(*t *+ 1) = ***A***·***z***(*t*) + ***Bu***(*t*) + ***n***_1_(*t*)

by minimizing the system noise ***n***_1_(*t*). This is equivalent to minimize the cost function

CF=∑j=1m‖z(tj)−v(tj)‖2
 MathType@MTEF@5@5@+=feaafiart1ev1aaatCvAUfKttLearuWrP9MDH5MBPbIqV92AaeXatLxBI9gBaebbnrfifHhDYfgasaacH8akY=wiFfYdH8Gipec8Eeeu0xXdbba9frFj0=OqFfea0dXdd9vqai=hGuQ8kuc9pgc9s8qqaq=dirpe0xb9q8qiLsFr0=vr0=vr0dc8meaabaqaciaacaGaaeqabaqabeGadaaakeaacqWGdbWqcqWGgbGrcqGH9aqpdaaeWaqaamaafmaabaacbmGae8NEaONaeiikaGIaemiDaq3aaSbaaSqaaiabdQgaQbqabaGccqGGPaqkcqGHsislcqWF2bGDcqGGOaakcqWG0baDdaWgaaWcbaGaemOAaOgabeaakiabcMcaPaGaayzcSlaawQa7aaWcbaGaemOAaOMaeyypa0JaeGymaedabaGaemyBa0ganiabggHiLdGcdaahaaWcbeqaaiabikdaYaaaaaa@482D@

where the time-variant vector ***v***(*t*) has the same dimensions as the internal state vector ***z***(*t *+ 1) and is calculated by the following difference equation

***v***(*t *+ 1) = ***A***·***v***(*t*) + ***Bu***(*t*)

with the initial state value ***v***(0) = ***z***(*t*_0_), and control values *u*(0),⋯,*u*(*t*).

For equally spaced measurements, the minimization of the cost function (10) can be solved by the least square method for the linear regression problem [55]. For unequally spaced measurements, the problem becomes nonlinear, and it is necessary to determine matrices ***A ***by using an optimization technique such as those in Chapter 10 of Press's text [56]. The matrix ***A ***contains *p*^2 ^unknown elements while the matrix ***Z ***contains *m*·*p *known expression data points. If *p *<*m*, matrix ***A ***can be uniquely determined. Fortunately, using AIC the number of chosen internal variables *p *generally is less than the numbers of genes *n *and time points *m*. Therefore matrices ***A ***in model (1) could unambiguously be estimated from time-course gene expression data.

#### Model evaluation

In this study, the inferred gene regulatory networks will be evaluated in the following aspects: the prediction power, stability, robustness, periodicity, controllability, and observability.

##### The *prediction power*

We use the two indices to measure the prediction power: The prediction error and the prediction correlation. Let X^
 MathType@MTEF@5@5@+=feaafiart1ev1aaatCvAUfKttLearuWrP9MDH5MBPbIqV92AaeXatLxBI9gBaebbnrfifHhDYfgasaacH8akY=wiFfYdH8Gipec8Eeeu0xXdbba9frFj0=OqFfea0dXdd9vqai=hGuQ8kuc9pgc9s8qqaq=dirpe0xb9q8qiLsFr0=vr0=vr0dc8meaabaqaciaacaGaaeqabaqabeGadaaakeaacuWHybawgaqcaaaa@2DF9@ be a data matrix with the same size as the original data matrix ***X***, which is computed from the model inferred from the data matrix ***X***. The prediction error reflects how well X^
 MathType@MTEF@5@5@+=feaafiart1ev1aaatCvAUfKttLearuWrP9MDH5MBPbIqV92AaeXatLxBI9gBaebbnrfifHhDYfgasaacH8akY=wiFfYdH8Gipec8Eeeu0xXdbba9frFj0=OqFfea0dXdd9vqai=hGuQ8kuc9pgc9s8qqaq=dirpe0xb9q8qiLsFr0=vr0=vr0dc8meaabaqaciaacaGaaeqabaqabeGadaaakeaacuWHybawgaqcaaaa@2DF9@ approximates **X**. The prediction error (*P*_*E*_) is defined as:

PE=1n∑i=1n‖X(i,:)−X^(i,:)‖2/‖X(i,:)‖2
 MathType@MTEF@5@5@+=feaafiart1ev1aaatCvAUfKttLearuWrP9MDH5MBPbIqV92AaeXatLxBI9gBaebbnrfifHhDYfgasaacH8akY=wiFfYdH8Gipec8Eeeu0xXdbba9frFj0=OqFfea0dXdd9vqai=hGuQ8kuc9pgc9s8qqaq=dirpe0xb9q8qiLsFr0=vr0=vr0dc8meaabaqaciaacaGaaeqabaqabeGadaaakeaacqWGqbaudaWgaaWcbaGaemyraueabeaakiabg2da9maalaaabaGaeGymaedabaGaemOBa4gaamaaqahabaWaaSGbaeaadaqbdaqaaGqadiab=HfayjabcIcaOiabdMgaPjabcYcaSiabcQda6iabcMcaPiabgkHiTiqb=HfayzaajaGaeiikaGIaemyAaKMaeiilaWIaeiOoaOJaeiykaKcacaGLjWUaayPcSdWaaWbaaSqabeaacqaIYaGmaaaakeaadaqbdaqaaiab=HfayjabcIcaOiabdMgaPjabcYcaSiabcQda6iabcMcaPaGaayzcSlaawQa7amaaCaaaleqabaGaeGOmaidaaaaaaeaacqWGPbqAcqGH9aqpcqaIXaqmaeaacqWGUbGBa0GaeyyeIuoaaaa@5585@

where ***X***(*i*,:) is the expression profile of gene *i *in the data matrix ***X***·||***X***(*i*,:)|| is the Euclidean norm of the vector ***X***(*i*,:). Intuitively, the smaller the prediction error, the stronger the prediction power is. The prediction error *P*_*E *_defined in (12) is invariant with respect to the scale of **X**. Therefore, it is more reasonable to evaluate the models using formulae (12) than the one defined by Wessels et al [57].

The prediction correlationerror (*P*_*C*_) is defined as:

PC=1n∑i=1ncor(X(i,:),X^(i,:))
 MathType@MTEF@5@5@+=feaafiart1ev1aaatCvAUfKttLearuWrP9MDH5MBPbIqV92AaeXatLxBI9gBaebbnrfifHhDYfgasaacH8akY=wiFfYdH8Gipec8Eeeu0xXdbba9frFj0=OqFfea0dXdd9vqai=hGuQ8kuc9pgc9s8qqaq=dirpe0xb9q8qiLsFr0=vr0=vr0dc8meaabaqaciaacaGaaeqabaqabeGadaaakeaacqWGqbaudaWgaaWcbaGaem4qameabeaakiabg2da9maalaaabaGaeGymaedabaGaemOBa4gaamaaqahabaGaem4yamMaem4Ba8MaemOCaiNaeiikaGccbmGae8hwaGLaeiikaGIaemyAaKMaeiilaWIaeiOoaOJaeiykaKIaeiilaWIaf8hwaGLbaKaacqGGOaakcqWGPbqAcqGGSaalcqGG6aGocqGGPaqkcqGGPaqkaSqaaiabdMgaPjabg2da9iabigdaXaqaaiabd6gaUbqdcqGHris5aaaa@4C8A@

where *cor*(*x,y*) is the standard correlation of two vectors [51]. The large prediction correlation indicates the strong prediction power.

##### Stability

Due to the limited energy and storage within a cell, concentrations of gene expression products such as mRNA should remain bounded. All real-life gene networks are therefore stable. Consequently, the inferred gene network models should also be stable in order to be realistic. For our model, this is equivalent to the governing equation (9) being stable. It has been proven [45] that the equation (9) is stable if and only if all eigenvalues of the state transition matrix *A *lie inside the unit circle in the complex plane.

##### Periodicity

Certain biological processes are periodic. The cell-cycle and circadian clock, for example, repeat at well-defined and reliable time intervals. Studies have shown that gene regulatory networks associated with these periodic biological processes are themselves rhythmic [58-60]. Therefore, the inferred gene regulatory networks associated with these periodic biological processes should be periodic at its stable states. Accordingly, the periodicity of system (1) at its stable state is determined by its dominant eigenvalues of the state transition matrix ***A ***whose moduli are the largest.

##### Robustness

The robustness of a gene regulatory network is understood as its insensitivity to noise or disturbance. It is obvious that a real-life gene regulatory network has robustness [47,58]. Therefore, the inferred gene regulatory network should be robust. The stability of a linear system implies robustness to a certain degree [46]. Note that the stability, robustness, and periodicity of system (1) are all related to the eigenvalues of the state transition matrix ***A***.

##### Controllability

A dynamic control system is said to be controllable if any state could be transferred to any preset state by appropriate control actions [46]. For example, if a gene regulatory network is controllable, it can always be transferred to a normal state if the network malfunctions and deviates from the normal state. The inferred network should be controllable if a real-life gene regulatory network is. It has been proven that the linear system (1) is controllable [46] if and only if

*rank*([*B*, *AB*, ⋯, *A*^*p*^*B*]) ≥ *p*

A control system is said to be directly controllable if *rank*(*B*) ≥ *p*. A directly controllable system can more easily transfer one state to the other one than a controllable system can. Because of the proposed modeling method in this study, all inferred gene regulatory networks are controllable.

##### Observability

A dynamic control system is said to be observable [46] if the internal state could be estimated by the observation data of the systems. For an observable gene regulatory network, one can always estimated its internal states from the observation data (i.e., time-course gene expression data) even though one can not directly "see" the behavior of the internal variables. Its inferred network should be observable if a real gene regulatory network is. Because of the proposed modeling method in this study, all inferred gene regulatory networks are observable.

## Authors' contributions

XH carried out the scale-free clustering approach. FXW carried out the state-space modelling and computational experiments. Both authors participated in design and coordination of the study, drafted the manuscript, and read and approved the final manuscript.

## Supplementary Material

Additional File 1 Prediction results of four complexes. Click here for file
