# Supplementary material for: Mining and state-space modeling and verification of sub-networks from large-scale biomolecular networks
Source: BMC Bioinformatics. 2007 Aug 31;8:324. doi: 10.1186/1471-2105-8-324 (PMC2213691; doi:10.1186/1471-2105-8-324)
Supplement: Additional File 1 — Prediction results of four complexes. [file 1471-2105-8-324-S1.doc]

This data file contains the comparison results of 4 complexes.

The first community, shown in Figure 1, is identified using TAF6 as seed. TAF6 is a component of the SAGA complex which is a multifunctional co-activator that regulates transcription by RNA polymerase II . The SAGA complex is listed in MIPS complex catalogue as a known cellular complex consisting of 16 proteins. As shown in Table 1, the community identified by our algorithm contains 39 members, including 14 of the 16 SAGA complex proteins listed in MIPS (indicated by an asterisk in the *Alias* column). The community also contains 14 of 21 proteins listed in MIPS as Kornberg’s mediator (SRB) complex. The rest of the proteins in the community are either TATA-binding proteins or transcription factor IID (TFIID) subunits or SRB related. TFIID is a complex involved in initiation of RNA polymerase II transcription. SAGA and TFIID are structurally and functionally correlated, make overlapping contributions to the expression of RNA polymerase II transcribed genes. SRB complex is a mediator that conveys regulatory signals from DNA-binding transcription factors to RNA polymerase II. In addition, 27 of the top 50 potential co-complex proteins (9 of the top 10), not including the seed proteins, predicted by Complexpander are in the identified community.


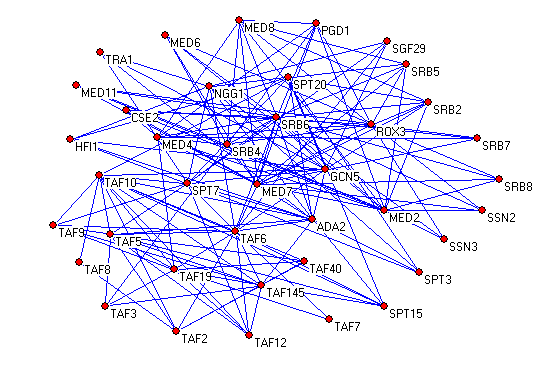


Fig. 1. The SAGA/SRB community.

TABLE I

The SAGA/SRB community: Proteins that belong to SAGA complex listed in MIPS complex catalogue database are indicated by (*) and those belonging to SRB complex are indicated by (†). Ranking is done by running Complexpander using the seed (TAF6) as the core protein set.

| *Protein* | *Alias* | *Description* | *Rank* |
| --- | --- | --- | --- |
| YDR448w | ADA2* | general transcriptional adaptor or co-activator | 1 |
| YNR010w | CSE2† | subunit of RNA polymerase II mediator complex |  |
| YGR252w | GCN5* | histone acetyltransferase | 2 |
| YPL254w | HFI1* | transcriptional coactivator | 3 |
| YMR112c | MED11† | mediator complex subunit |  |
| YDL005c | MED2† | transcriptional regulation mediator | 20 |
| YOR174w | MED4† | transcription regulation mediator | 23 |
| YHR058c | MED6† | RNA polymerase II transcriptional regulation mediator |  |
| YOL135c | MED7† | member of RNA Polymerase II transcriptional regulation mediator complex | 21 |
| YBR193c | MED8† | transcriptional regulation mediator | 24 |
| YDR176w | NGG1* | general transcriptional adaptor or co-activator | 10 |
| YGL025c | PGD1† | mediator complex subunit | 37 |
| YBL093c | ROX3† | transcription factor |  |
| YCL010c | SGF29* | SAGA associated factor | 43 |
| YER148w | SPT15 | the TATA-binding protein TBP | 15 |
| YOL148c | SPT20* | member of the TBP class of SPT proteins that alter transcription site selection | 4 |
| YDR392w | SPT3* | general transcriptional adaptor or co-activator | 13 |
| YBR081c | SPT7* | involved in alteration of transcription start site selection | 5 |
| YHR041c | SRB2† | DNA-directed RNA polymerase II holoenzyme and Kornberg^s mediator (SRB) subcomplex subunit |  |
| YER022w | SRB4† | DNA-directed RNA polymerase II holoenzyme and Kornberg^s mediator (SRB) subcomplex subunit | 27 |
| YGR104c | SRB5† | DNA-directed RNA polymerase II holoenzyme and Kornberg^s mediator (SRB) subcomplex subunit |  |
| YBR253w | SRB6† | DNA-directed RNA polymerase II suppressor protein | 19 |
| YDR308c | SRB7† | DNA-directed RNA polymerase II holoenzyme and kornberg^s mediator (SRB) subcomplex subunit | 46 |
| YCR081w | SRB8 | DNA-directed RNA polymerase II holoenzyme and Srb10 CDK subcomplex subunit |  |
| YDR443c | SSN2 | DNA-directed RNA polymerase II holoenzyme and Srb10 CDK subcomplex subunit |  |
| YPL042c | SSN3 | cyclin-dependent CTD kinase |  |
| YGR274c | TAF1 | TFIID subunit (TBP-associated factor), 145 kD | 14 |
| YDR167w | TAF10* | TFIID and SAGA subunit | 7 |
| YML015c | TAF11 | TFIID subunit (TBP-associated factor), 40KD | 18 |
| YDR145w | TAF12* | TFIID and SAGA subunit | 8 |
| YML098w | TAF13 | TFIID subunit (TBP-associated factor), 19 kD | 17 |
| YCR042c | TAF2 | component of TFIID complex | 22 |
| YPL011c | TAF3 | component of the TBP-associated protein complex | 50 |
| YBR198c | TAF5* | TFIID and SAGA subunit | 9 |
| YGL112c | TAF6* | TFIID and SAGA subunit |  |
| YMR227c | TAF7 | TFIID subunit (TBP-associated factor), 67 kD |  |
| YML114c | TAF8 | TBP Associated Factor 65 KDa |  |
| YMR236w | TAF9* | TFIID and SAGA subunit | 11 |
| YHR099w | TRA1* | component of the Ada-Spt transcriptional regulatory complex | 12 |

The second community is discovered using NOT3 as seed (Figure 2). NOT3 is a known component protein of the CCR4-NOT complex which is a global regulator of gene expression and involved in such functions as transcription regulation and DNA damage responses. MIPS complex catalogue lists 5 proteins for NOT complex and 13 proteins (including the 5 NOT complex proteins) for CCR4 complex. The NOT community identified is composed of 40 members. All 5 NOT complex proteins listed in MIPS and 11 of the 13 CCR4 complex proteins are members of the community. POL1, POL2, PRI1, and PRI2 are members of the DNA polymerase alpha (I) – primase complex, as listed in MIPS. RVB1, PIL1, UBR1, and STI1 have been grouped together with CCR4, CDC39, CDC36, and POP2 by systematic analysis. The community also contains 20 out of 26 proteins of a complex that probably is involved in transcription and DNA/chromatin structure maintenance.


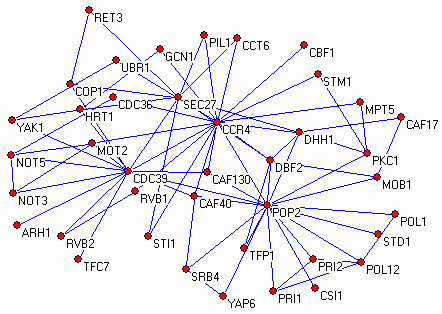


Fig. 2. The CCR4-NOT community.

Table II

The CCR4-NOT community: Proteins belonging to CCR4-NOT complex listed in MIPS are indicated by (*) and proteins considered to be involved in transcription and DNA/chromatin structure maintenance are indicate by (†).

| *Protein* | *Alias* | *Description* | *Rank* |
| --- | --- | --- | --- |
| YDR376w | ARH1 | mitochondrial protein putative ferredoxin-NADP+ reductase | 38 |
| YGR134w | CAF130† | CCR4 Associated Factor 130 kDa | 8 |
| YJR122w | CAF17* | CCR4 associated factor |  |
| YNL288w | CAF40† | CCR4 Associated Factor 40 kDa | 9 |
| YJR060w | CBF1 | centromere binding factor 1 |  |
| YAL021c | CCR4*† | transcriptional regulator | 3 |
| YDR188w | CCT6† | component of chaperonin-containing T-complex (zeta subunit) | 30 |
| YDL165w | CDC36*† | transcription factor | 40 |
| YCR093w | CDC39*† | nuclear protein | 1 |
| YDL145c | COP1† | coatomer complex alpha chain of secretory pathway vesicles | 11 |
| YMR025w | CSI1 | Subunit of the Cop9 signalosome, involved in adaptation to pheromone signaling | 46 |
| YGR092w | DBF2* | ser/thr protein kinase related to Dbf20p | 6 |
| YDL160c | DHH1* | DExD/H-box helicase, stimulates mRNA decapping, | 17 |
| YGL195w | GCN1† | translational activator | 26 |
| YOL133w | HRT1 | Skp1-Cullin-F-box ubiquitin protein ligase (SCF) subunit |  |
| YIL106w | MOB1* | required for completion of mitosis and maintenance of ploidy | 10 |
| YER068w | MOT2*† | transcriptional repressor | 2 |
| YGL178w | MPT5 | multicopy suppressor of POP2 |  |
| YIL038c | NOT3*† | general negative regulator of transcription, subunit 3 |  |
| YPR072w | NOT5*† | component of the NOT protein complex | 5 |
| YGR086c | PIL1 | Long chain base-responsive inhibitor of protein kinases Phk1p and Phk2p, acts along with Lsp1p to down-regulate heat stress resistance |  |
| YBL105c | PKC1 | ser/thr protein kinase |  |
| YNL102w | POL1† | DNA-directed DNA polymerase alpha, 180 KD subunit | 32 |
| YBL035c | POL12† | DNA-directed DNA polymerase alpha, 70 KD subunit | 28 |
| YNR052c | POP2*† | required for glucose derepression | 4 |
| YIR008c | PRI1† | DNA-directed DNA polymerase alpha 48kDa subunit (DNA primase) | 34 |
| YKL045w | PRI2† | DNA-directed DNA polymerase alpha , 58 KD subunit (DNA primase) | 31 |
| YPL010w | RET3 | coatomer complex zeta chain | 39 |
| YDR190c | RVB1 | RUVB-like protein | 29 |
| YPL235w | RVB2† | RUVB-like protein | 21 |
| YGL137w | SEC27† | coatomer complex beta^ chain (beta^-cop) of secretory pathway vesicles | 7 |
| YER022w | SRB4 | DNA-directed RNA polymerase II holoenzyme and Kornberg^s mediator (SRB) subcomplex subunit | 44 |
| YOR047c | STD1 | dosage-dependent modulator of glucose repression |  |
| YOR027w | STI1 | stress-induced protein |  |
| YLR150w | STM1 | specific affinity for guanine-rich quadruplex nucleic acids |  |
| YOR110w | TFC7† | TFIIIC (transcription initiation factor) subunit, 55 kDa | 25 |
| YDL185w | TFP1† | encodes 3 region protein which is self-spliced into TFP1p and PI-SceI | 27 |
| YGR184c | UBR1 | ubiquitin-protein ligase |  |
| YJL141c | YAK1 | ser/thr protein kinase |  |
| YDR259c | YAP6 | transcription factor, of a fungal-specific family of bzip proteins |  |

The third community is identified by using RFC2 as the seed (Figure 3). RFC2 is a component of the RFC (replication factor C) complex, the “clamp loader”, which plays an essential role in DNA replication and DNA repair. The community identified by our algorithm has 17 members. All five proteins of RFC complex listed in MIPS complex catalogue database are members of this community, as shown in Table 3. All but one member in this community are in the functional category of DNA recombination and DNA repair or cell cycle checkpoints according to MIPS. This community also includes the top 8 ranked proteins predicted by Complexpander.


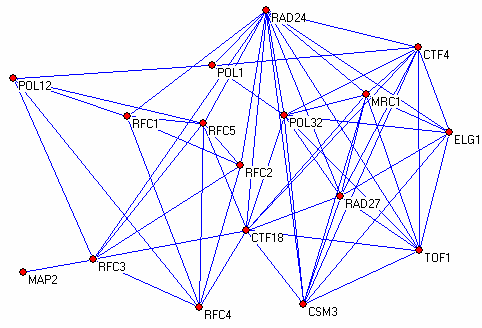


Fig. 3. The RFC community.

Table III

The RFC community. Proteins belonging to RFC complex listed in MIPS are indicated by (*) and proteins listed in the functional category of DNA recombination and DNA repair or cell cycle checkpoints by MIPS are indicated by (†).

| *Protein* | *Alias* | *Description* | *Rank* |
| --- | --- | --- | --- |
| YMR048w | CSM3† | Protein required for accurate chromosome segregation during meiosis |  |
| YMR078c | CTF18† | required for accurate chromosome transmission in mitosis and maintenance of normal telomere length | 6 |
| YPR135w | CTF4† | DNA-directed DNA polymerase alpha-binding protein |  |
| YOR144c | ELG1† | Protein required for S phase progression and telomere homeostasis, forms an alternative replication factor C complex important for DNA replication and genome integrity | 7 |
| YBL091c | MAP2 | methionine aminopeptidase, isoform 2 |  |
| YCL061c | MRC1† | Mediator of the Replication Checkpoint |  |
| YNL102w | POL1† | DNA-directed DNA polymerase alpha, 180 KD subunit | 19 |
| YBL035c | POL12† | DNA-directed DNA polymerase alpha, 70 KD subunit | 5 |
| YJR043c | POL32† | polymerase-associated gene, third (55 kDa) subunit of DNA polymerase delta |  |
| YER173w | RAD24† | cell cycle checkpoint protein | 1 |
| YKL113c | RAD27† | ssDNA endonuclease and 5^-3^exonuclease |  |
| YOR217w | RFC1*† | DNA replication factor C, 95 KD subunit | 8 |
| YJR068w | RFC2*† | DNA replication factor C, 41 KD subunit |  |
| YNL290w | RFC3*† | DNA replication factor C, 40 kDa subunit | 2 |
| YOL094c | RFC4*† | DNA replication factor C, 37 kDa subunit | 4 |
| YBR087w | RFC5*† | DNA replication factor C, 40 KD subunit | 3 |
| YNL273w | TOF1† | topoisomerase I interacting factor 1 |  |

We use ARP3 as seed to identify the last community (Figure 4). ARP2/ARP3 complex acts as multi-functional organizer of actin filaments. The assembly and maintenance of many actin-based cellular structures likely depend on functioning ARP2/ARP3 complex. The identified community contains all 7 proteins of the ARP2/ARP3 complex listed in MIPS (Table 4). Not including the seed (ARP3), these proteins represent the top 6 ranked proteins predicted by Complexpander. As indicated in Table 4, there are 14 members belonging to the same functional category of budding, cell polarity, and filament formation, according to MIPS.


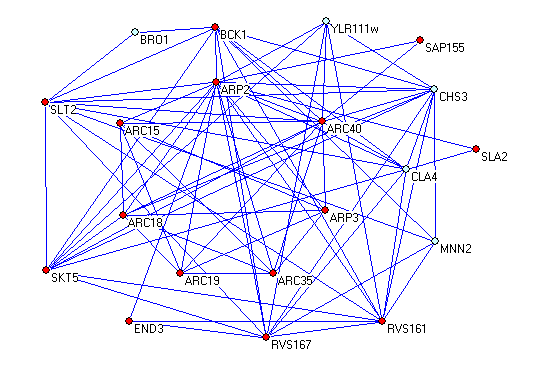


Fig. 4 The ARP2/ARP3 community.

Table IV

The ARP2/ARP3 community. Proteins belonging to ARP2/3 complex listed in MIPS are indicated by (*) and proteins listed in the functional category of budding, cell polarity, and filament formation by MIPS are indicated by (†).

| Protein | *Alias* | *Description* | *Rank* |
| --- | --- | --- | --- |
| YLR111w | YLR111w | hypothetical protein |  |
| YIL062c | ARC15*† | subunit of the Arp2/3 complex | 1 |
| YLR370c | ARC18* | subunit of the Arp2/3 complex | 4 |
| YKL013c | ARC19*† | subunit of the Arp2/3 complex | 3 |
| YNR035c | ARC35* | subunit of the Arp2/3 complex | 5 |
| YBR234c | ARC40*† | Arp2/3 protein complex subunit, 40 kilodalton | 6 |
| YDL029w | ARP2*† | actin-like protein | 2 |
| YJR065c | ARP3* | actin related protein |  |
| YJL095w | BCK1† | ser/thr protein kinase of the MEKK family |  |
| YPL084w | BRO1 | required for normal response to nutrient limitation |  |
| YBR023c | CHS3† | chitin synthase III |  |
| YNL298w | CLA4† | ser/thr protein kinase |  |
| YNL084c | END3† | required for endocytosis and cytoskeletal organization |  |
| YBR015c | MNN2 | type II membrane protein |  |
| YCR009c | RVS161† | protein involved in cell polarity development |  |
| YDR388w | RVS167† | reduced viability upon starvation protein |  |
| YFR040w | SAP155† | Sit4p-associated protein |  |
| YBL061c | SKT5† | protoplast regeneration and killer toxin resistance protein |  |
| YNL243w | SLA2† | cytoskeleton assembly control protein |  |
| YHR030c | SLT2† | ser/thr protein kinase of MAP kinase family |  |
